# A Nonsense Mutation in the *IKBKG* Gene in Mares with Incontinentia Pigmenti

**DOI:** 10.1371/journal.pone.0081625

**Published:** 2013-12-04

**Authors:** Rachel E. Towers, Leonardo Murgiano, David S. Millar, Elise Glen, Ana Topf, Vidhya Jagannathan, Cord Drögemüller, Judith A. Goodship, Angus J. Clarke, Tosso Leeb

**Affiliations:** 1 Institute of Medical Genetics, Cardiff University, Cardiff, United Kingdom; 2 Institute of Genetic Medicine, Newcastle University, Newcastle upon Tyne, United Kingdom; 3 Institute of Genetics, Vetsuisse Faculty, University of Bern, Bern, Switzerland; 4 DermFocus, Vetsuisse Faculty, University of Bern, Bern, Switzerland; Università degli Studi di Milano, Italy

## Abstract

Ectodermal dysplasias (EDs) are a large and heterogeneous group of hereditary disorders characterized by abnormalities in structures of ectodermal origin. Incontinentia pigmenti (IP) is an ED characterized by skin lesions evolving over time, as well as dental, nail, and ocular abnormalities. Due to X-linked dominant inheritance IP symptoms can only be seen in female individuals while affected males die during development *in utero*. We observed a family of horses, in which several mares developed signs of a skin disorder reminiscent of human IP. Cutaneous manifestations in affected horses included the development of pruritic, exudative lesions soon after birth. These developed into wart-like lesions and areas of alopecia with occasional wooly hair re-growth. Affected horses also had streaks of darker and lighter coat coloration from birth. The observation that only females were affected together with a high number of spontaneous abortions suggested an X-linked dominant mechanism of transmission. Using next generation sequencing we sequenced the whole genome of one affected mare. We analyzed the sequence data for non-synonymous variants in candidate genes and found a heterozygous nonsense variant in the X-chromosomal *IKBKG* gene (c.184C>T; p.Arg62*). Mutations in *IKBKG* were previously reported to cause IP in humans and the homologous p.Arg62* variant has already been observed in a human IP patient. The comparative data thus strongly suggest that this is also the causative variant for the observed IP in horses. To our knowledge this is the first large animal model for IP.

## Introduction

Ectodermal dysplasias (EDs) are a large and heterogeneous group of hereditary disorders characterized by abnormalities in multiple structures of ectodermal origin. Approximatively 200 different conditions of ED are known in humans. [1]–[3]. Outside humans, spontaneous forms of EDs have been reported extensively in cattle [4]–[7] and dogs [8]–[10].

EDs are caused by mutations in genes belonging to different pathways. There are more than 30 genes associated with EDs. Many of these genes are in the ectodyplasin, NFκB, and WNT pathways [1]–[3], [11], [12]. The NFκB family comprises transcription factors involved in the regulation of diverse cellular processes, including inflammation, innate/adaptive immunity, and cell survival during development [13]. In nonstimulated cells, some NFκB transcription factors are bound to inhibitory IκB proteins and are thereby sequestered in the cytoplasm. Activation occurs upon phosphorylation and subsequent degradation of the IκB complex. Two protein kinases with a high degree of sequence similarity, IKKα (approved symbol: CHUK) and IKKβ (approved symbol: IKBKB), mediate phosphorylation of IκB proteins and represent a convergence point for most signal transduction pathways leading to NFκB activation. Most of the IKK complexes also contain a regulatory subunit called IKKγ or NFκB essential modulator (NEMO), which is encoded by the X-chromosomal *IKBKG* gene [13].

Mutations in the *IKBKG* gene can lead to various, clinically distinct EDs in humans. Hypomorphic mutations typically affecting the C-terminal region of the encoded protein lead to the X-chromosomal recessive hypohidrotic or anhidrotic ectodermal dysplasia with immune deficiency (HED-ID, OMIM #300291) [14]. Other rare types of mutations in the *IKBKG* gene lead to isolated immune deficiencies (OMIM #300584, #300636, and #300640) [15]. Most known variants including the presumed complete loss-of-function variants in the human *IKBKG* gene lead to an ED with characteristic clinical features termed incontinentia pigmenti (IP, OMIM #308300) [16], [17].

IP in humans is a rare disorder of several ectodermal tissues including hair, skin, teeth, nails, and eyes. The condition is transmitted as an X-linked dominant trait with intrauterine lethality of the affected, hemizygous males. Females heterozygous for *IKBKG* mutations have clinical features of IP and demonstrate skewed X-inactivation. An increased risk of cell death in cells expressing the abnormal *IKBKG* gene results in negative selection, leaving more cells expressing the wild type IKBKG to survive and proliferate. This is reflected in the clinical course of IP, where patients show skin blistering and severe skin lesions immediately after birth. In later stages, most cells expressing the mutant X-chromosome will be eliminated and the skin symptoms largely resolve. Adult IP patients show characteristic patterns of hyperpigmentation, which follow Blaschko's lines [18]. A number of mutations in *IKBKG* have been identified to cause IP in humans. The most common mutation is a deletion of exons 4 to 10, which are flanked by two MER67B repeat sequences [16], [17].

We have identified horses that show a phenotype with many similarities to human IP. The aim of this study was to elucidate the underlying genetic defect in these horses.

## Results

### Phenotypic description

**Figure 1:**
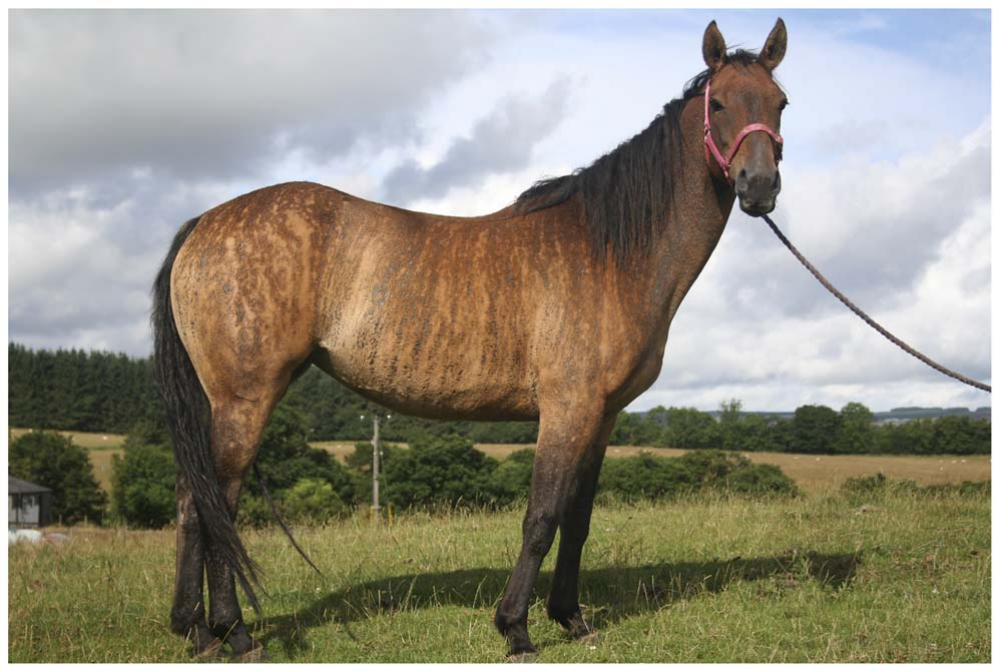
Phenotype of an affected horse. Brindled pigmentation and patches of hairless skin are visible. The hairless skin probably represents scarring alopecia following the different stages of skin lesions described. The patterns of hyperpigmentation and the skin alterations follow the lines of Blaschko. The depicted horse corresponds to animal III-10 in the pedigree shown in Figure 2.

We observed a family of horses, in which several mares developed signs of a skin disorder reminiscent of human IP. Cutaneous manifestations in affected horses included the development of pruritic, exudative lesions soon after birth. These developed into wart-like lesions and areas of alopecia. Occasionally, we observed hair re-growth with a wooly appearance. Affected horses also had streaks of darker and lighter coat coloration from birth. These cutaneous manifestations followed the lines of Blaschko. Other clinical symptoms included anomalies of tooth, hoof and ocular development (Figure 1).

### Pedigree analysis

**Figure 2:**
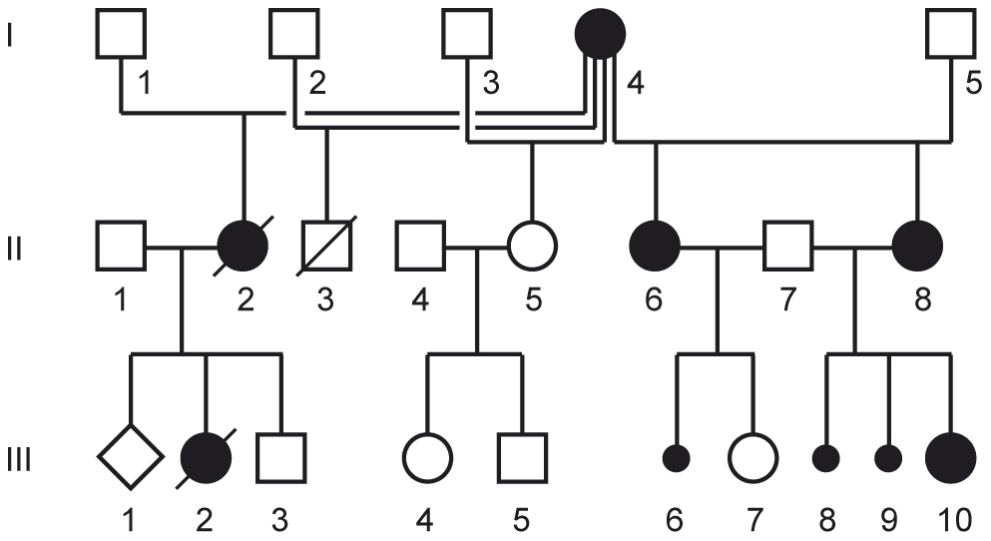
Pedigree of the horse family presented in this study. The affected animals are shown as filled symbols. Non-affected animals are shown with open symbols. Males are shown as squares and females as circles; aborted foals (sex unknown) are shown as small black circles. The whole genome re-sequencing experiment was performed with DNA from animal II-8. The horse II-2 was killed in an accident and III-2 was born dead. Both deceased animals showed clear signs of IP.

All the affected mares belonged to the same family and were descendants of one affected founder mare. All affected animals were female, and two of them had reported abortions. Thus, the pedigree was compatible with an X-chromosomal dominant mode of inheritance (Figure 2).

### Mutation identification

We sequenced the whole genome of one affected mare in order to get a comprehensive overview of all sequence variants (animal II-8 in Figure 2). We collected 225 million 2×100 bp paired-end reads from a shotgun fragment library corresponding to roughly 19× coverage of the genome. We called SNPs and indel variants with respect to the EquCab 2 reference genome and identified approximately 7.8 million variants in total. The observed phenotype and alleged mechanism of transmission of the condition prompted us to focus exclusively on heterozygous variants on the X chromosome. The data contained 557 X-chromosomal non-synonymous variants. We hypothesized that the mutant allele at the causative variant should be completely absent from the general horse population. Therefore, we compared the variants in the IP affected horse with the genomes of 44 control horses from 11 breeds that had been sequenced in the course of other projects. This filtering step resulted in 33 private and non-synonymous X-chromosomal variants in the IP affected horse (Table 1).

**Table 1:** Summary of the whole genome sequencing experiment.

Filtering step	Number of variants
Total variants in the whole genome^a^	7,807,149
Heterozygous variants in the whole genome	4,969,029
Heterozygous variants on the X-chromosome	191,844
Non-synonymous heterozygous variants on the X-chromosome	557
Non-synonymous heterozygous variants on the X-chromosome that were absent from 44 other horse genomes	33

a Variants were called with respect to the reference genome (EquCab 2) from a Thoroughbred horse.

**Figure 3:**
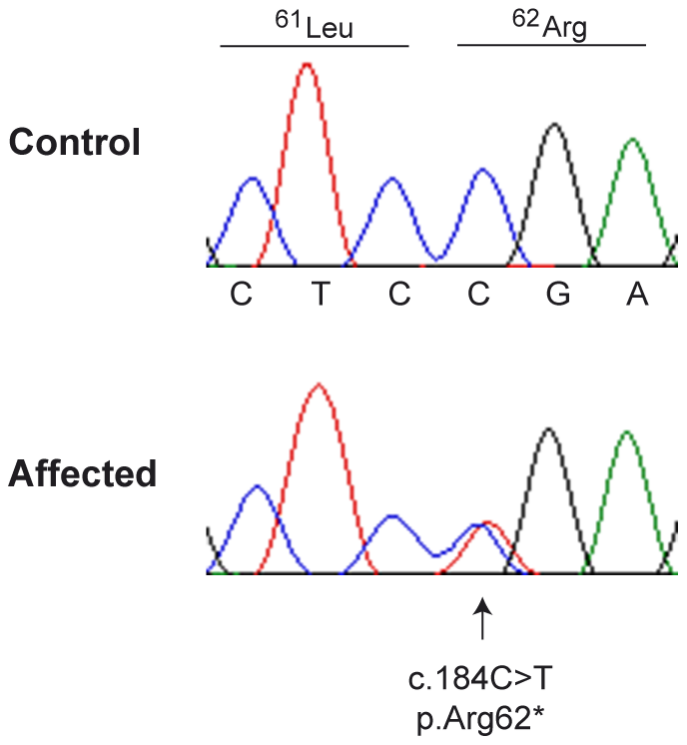
Sanger sequencing of the *IKBKG:c.184C>T* variant. Electropherograms of a homozygous wildtype and a heterozygous mare are shown. The reading frame and position of the nonsense variant are indicated. The variant is chrX:122,833,887C>T with respect to the EquCab 2 genome reference sequence.

We screened these results for variants in plausible functional candidate genes and noticed a heterozygous c.184C>T mutation in the *IKBKG* gene. This variant is a nonsense variant, predicted to result in a premature stop codon, which truncates more than 85% of the protein (p.Arg62*). We confirmed the co-segregation of the variant with the phenotype in 3 affected and one non-affected horse of the family by Sanger sequencing (Figure 3).

### Transcript analysis

The equine *IKBKG* mRNA predictions from the NCBI and ENSEMBL databases were significantly different from each other and also did not align well with the human *IKBKG* mRNA sequence. Therefore, we determined an experimental equine *IKBKG* mRNA sequence from an RT-PCR product. We deposited this sequence in the EMBL/Genbank/DDBJ databases under accession number KF471022. Aligning this sequence to the EquCab 2 reference genome sequence confirmed that the equine *IKBKG* gene is similar to the other known mammalian orthologs and contains nine coding exons spread over ∼14 kb of genomic DNA on the X chromosome. Exon 4 lies within an unsequenced gap region of genomic DNA and its exact position could not be ascertained. All available exon/intron boundaries conformed to the AG/GT splicing consensus sequences (Table 2).

**Table 2:** Exons of the equine *IKBKG* gene.

Exon^a^	Start^b^	Stop^b^	Length (bp)	Prec. intron	Exon start	Exon end	Subseq. intron
							
2	<122,833,704	122,833,890	>187		n.d.	CCGAG	gtgag
3	122,837,191	122,837,402	212	gccag	ATGCC	AGCAG	gtagc
4	n.d.	n.d.	119	n.d.	CAGAT	GGCAG	n.d.
5	122,844,285	122,844,437	153	gccag	GGCCC	GAGAA	gtgag
6	122,845,008	122,845,104	97	cttag	GCGGA	ACCGA	gtgag
7	122,846,220	122,846,363	144	tccag	GGAAT	CCCAG	gtgag
8	122,846,643	122,846,785	143	gccag	GCGGA	GCCAG	gtggg
9	122,846,995	122,847,056	62	ctaag	GATCG	CCCAG	gtgag
10	122,847,296	>122,847,438	>143	cccag	CTCAC	n.d.	

a The numbering of the exons corresponds to the situation in other mammals, which have an additional 5′-untranslated exon.

b The coordinates correspond to the X-chromosome of the EquCab 2.0 genome reference assembly.

Comparison of the equine *IKBKG* mRNA to other species showed high levels of homology between a number of different species ranging from 90% identity with *Bos taurus* and *Homo sapiens* to 96% with the Southern white rhinoceros (*Ceratotherium simum simum*). Similarly, the predicted protein sequence showed a high level of identity between species: 90% with *Bos taurus*, 92% with *Homo sapiens* and 97% with *Ceratotherium simum simum*.

## Discussion

In this study, we identified an *IKBKG* nonsense mutation in a family of horses perfectly co-segregating with a phenotype resembling human IP. The most recognizable symptoms of IP are skin lesions evolving over time. In our horses, we early on observed the erythema and vesicles, and the verrucous hyperkeratotic papules typical of preliminary phases of the disease (“phase 1” and “phase 2” [19]). In adult animals the whorls and streaks of pigmentation following the lines of Blaschko and pale, hairless, atrophic patches and/or hypopigmentation were visible, which corresponded well to “phase 3” and “phase 4” of human IP [19]. The X-chromosomal dominant inheritance of the equine IP phenotype further supported our hypothesis that the equine phenotype was genetically homologous to human IP.

Using a whole genome sequencing approach we detected almost 8 million variants in an affected horse compared to the equine genome reference sequence. By focusing on private X-chromosomal heterozygous non-synonymous variants, the initial daunting number of variants shrank to a much more manageable 33.

Only one of these variants was located in a plausible candidate gene for IP, the *IKBKG* gene. Mutations in the *IKBKG* gene have been shown to result in an IP phenotype in humans and mice [16], [20], [21]. In humans a large number of different mutations causing IP in humans have been reported [22]–[26]. A frequent recurrent deletion of exons 4 to 10 was found to account for more than 80% of all human IP cases [16], [17], [23]. An identical mutation to that identified in the studied family of horses has also been identified in a human patient with IP [17]. The c.184C>T transition identified in both cases occurs within a CpG dinucleotide. Transitions within the CpG dinucleotide account for approximately 23% of all single base pair substitutions causing human genetic disease [27] and are thought to occur due to spontaneous deamination of the methylated cytosine within the dinucleotide. It is likely that the mutation found in our horse family occurred by the same mechanism. Although we have not perfomed any functional validation of the equine variant, the human-horse comparative data strongly suggest that we indeed identified the causative variant for the observed phenotype in horses and the genetic findings further confirm that this is indeed an equine form of IP truly homologous to the human condition

In retrospect, we have to admit that we were lucky with our methodological approach. As the horse genome reference sequence and its annotation are in a preliminary draft status, there are many genes including the *IKBKG* gene, which are currently not correctly annotated. If the causative variant in the IP affected horses had been located e.g. in exon 4, which is currently not contained in the genomic reference sequence or in one of the later exons, which are not all correctly annotated, our whole genome sequencing experiment would have failed to yield the causative variant. This clearly emphasizes the need for continuous updating and improving of genome reference sequences, which are an enormously important asset in current veterinary genetics.

In conclusion, we have identified a nonsense variant in the equine *IKBKG* gene as most likely causative for a hereditary ectodermal dysplasia in horses. A mutation event at the homologous nucleotide position has independently occurred in a human family segregating for IP. Thus, the genetic findings in humans and horses mutually corroborate the causality of the variant and confirm that horses with this genetic defect are a valuable large animal model for human IP.

## Materials and Methods

### Ethics statement

All animal experiments were performed according to the local regulations. The horses in this study were examined with the consent of their owners. The experiments were approved by the ethical review committee of Newcastle University (ID 272).

### Animals

We used 3 female IP cases (II-6, II-8, III-10) and one female control horse (III-7) from the family depicted in Figure 2. Phenotypes were assigned by visual inspection of the skin pigmentation and hair quality. We additionally used 44 unrelated horses from 11 breeds as controls in this study. We isolated genomic DNA samples from hair roots or EDTA blood samples with the Nucleon Bacc2 kit (GE Healthcare).

### Whole genome sequencing of an affected mare

We prepared a fragment library with 300 bp insert size from animal II-8 (Figure 2) and collected one lane of illumina HiSeq2000 paired-end reads (2×100 bp). We obtained a total of 453,107,102 reads or roughly 19× coverage. We mapped the reads to the Equcab 2.0 reference genome with the Burrows-Wheeler Aligner (BWA) version 0.5.9-r16 [28] with default settings and obtained 440,799,216 (92.8%) uniquely mapping reads. After sorting the mapped reads by the coordinates of the sequence with Picard tools, we labeled the PCR duplicates also with Picard tools (http://sourceforge.net/projects/picard/). We used the Genome Analysis Tool Kit (GATK version 0591, [29]) to perform local realignment and to produce a cleaned BAM file. Variant calls were then made with the unified genotyper module of GATK. For variant calling we used only reads with mapping quality of ≥30 and bases with quality values ≥20. The variant data output file obtained in VCF format 4.0 was filtered for high quality SNPs using the variant filtering module of GATK. The filtering was done as explained in the GATK best practice manual 3.0. The snpEFF software [30] together with the EquCab 2.0 annotation was used to predict the functional effects of detected variants.

### Sanger sequencing

We used Sanger sequencing to confirm the illumina sequencing results and to verify the association of the mutation within family. For these experiments we amplified PCR products using AmpliTaqGold360Mastermix (Applied Biosystems). PCR products were directly sequenced on an ABI 3730 capillary sequencer (Applied Biosystems) after treatment with exonuclease I and shrimp alkaline phosphatase. We analyzed the sequence data with Sequencher 5.1 (GeneCodes).

### Gene analysis

The human *IKBKG* transcript variant 3 mRNA (accession: NM_003639.3) was used as query in cross-species BLAST searches against the horse genome assembly. Two publicly available equine *IKBKG* mRNA predictions show striking differences to the human sequence and are most likely not correct (LOC100058432, accession XM_001495456.3; ENSECAT00000022050.1). We therefore determined an experimental equine mRNA sequence (see above), which is available under accession KF471022. The genomic structure of the equine *IKBKG* gene was determined by aliging this experimental mRNA sequence to the EquCab 2.0 reference genome assembly using the Spidey program (www.ncbi.nlm.nih.gov/spidey).

### Transcript analysis

A skin biopsy from a control horse was finely minced and homogenized using the FastPrep system and lysing matrix D (MP Biomedical). Total RNA was then extracted using Trizol, DNase treated and quantified. One µg total RNA was then reverse transcribed using an oligo (dT)_25_ primer and Superscript II (Life Technologies) according to the manufacturer's instructions. PCR was then performed on the cDNA using oligonucleotide primers NEMO5 (5′-ACCCTGACTTGTTGGATGAGC-3′) and NEMO3 (5′-ACAGGCAGCCCTACTCGATG-3′) and the High Fidelity PCR system (Roche) using the following PCR cycle: 95°C 2 min followed by 35 cycles of 95°C 45 sec, 60°C 30 sec, 72°C 2 min followed by a final extension of 10 min at 72°C. The resulting PCR product was then sequenced with the same oligonucleotide primers used for PCR and the additional primers, NEMOSEQ1 (5′-ACGTGCTGGGTGAAGAGTC-3′), NEMOSEQ1R (5′-CCAGACAACGCTGGAAGG-3′) and NEMOSEQ2 (5′-ACGTGCAGGTGGACCAGC-3′) using BigDye v3.1 (Applied Biosystems) and sequenced on an ABI 3100 genetic analyser (Applied Biosystems).
